# The Role of Actin‐Filament Bundling Protein, Fimbrin Sac6, and Type‐V Myosin Myo2, in the Motion of the SUN‐Domain Protein, Mps3, During Yeast Meiosis

**DOI:** 10.1111/gtc.70156

**Published:** 2026-10-28

**Authors:** Shuangping Cheng, Tadasu Nozaki, Yurika Fujita, Masaru Ito, Akira Shinohara

**Affiliations:** ^1^ Institute for Protein Research, the University of Osaka Suita, Osaka Japan; ^2^ Department of Biology University of Massachusetts Amherst Amherst Massachusetts USA; ^3^ School of Basic Medical Sciences, Shenzhen University Shenzhen China

**Keywords:** actin cables, meiosis; nuclear envelope, Mps3, Myo2, Sac6

## Abstract

During meiosis, telomere clustering and motion promote the pairing between homologous chromosomes. In budding yeast, actin filaments in the cytoplasm drive the motion of a Linker Nucleoskeleton and Cytoskeleton (LINC) complex with the SUN‐domain protein, Mps3, which tethers and moves telomeres to the nuclear envelope (NE). In this study, we analyzed the roles of two actin‐binding proteins, essential Type‐V myosin (Myo2) and Sac6 fimbrin, which bundles actin filaments in cables, in meiotic Mps3 motion on the NE. Meiosis‐specific depletion of *MYO2* reduced the motion of Mps3 patches without affecting cytoplasmic actin cables during meiotic prophase I, confirming actin/myosin‐dependent Mps3 motion. Moreover, Myo2 plays a critical role in the maintenance of Mps3 clustering and, probably, telomere clustering in the absence of Rec8, a component of the meiotic cohesin. The deletion of *SAC6*, which is critical for force generation in the cytoplasm, during meiosis largely reduced cytoplasmic actin cables without affecting Mps3 localization and motion. Likely, Sac6‐mediated actin filament bundling is not critical for the meiotic motion of Mps3 patches. Our study suggests that actin polymerization and Myo2‐mediated force are involved in the meiotic motion of the yeast LINC complex.

## Graphical abstract

Type‐V myosin (Myo2) promotes the motion of Mps3 patches on the nuclear membrane during yeast meiosis. On the other hand, Sac6 fimbrin, which bundles actin filaments, is not critical for the motion of Mps3, suggesting the role of a few actin filaments in the Mps3 motion.

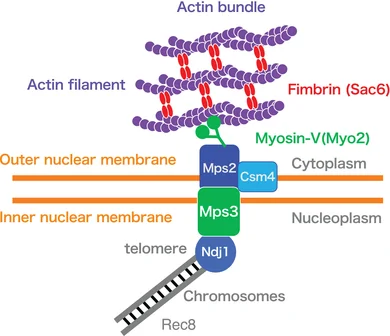

## 1 Introduction

During Prophase I of meiosis, telomeres, the ends of chromosomes, tether to and move on the nuclear envelope (NE). Telomeres cluster near the microtubule‐organizing center (MTOC) in middle Prophase I, referred to as the telomere bouquet (Hiraoka and Dernburg 2009). Telomere clustering during meiosis is highly conserved across organisms, including fission yeast, maize, mouse, human, and others (Chikashige et al. 1994; Sato et al. 2009; Scherthan et al. 1996) (Armstrong et al. 2001; Bass et al. 1997). After formation in mid‐prophase I, telomere clustering is resolved in late Prophase I.

Cytoskeleton‐driven forces promote telomere motion and clustering on the NE. The telomeres are tethered to an inner nuclear membrane (INM) protein via the SUN (Sad1/UNc‐84) domain. The SUN domain forms a trimer in the lumen (or perinuclear space) between INM and the outer nuclear membrane (ONM), and binds to a C‐terminal KASH (Klarsicht, ANC‐1, SYNE1 Homology) domain of the ONM protein (Gurusaran and Davies 2021; Sosa et al. 2012). The N‐terminal cytoplasmic region of the KASH‐domain protein binds to various cytoskeletons. The NE ensemble, which physically connects chromosomes or nuclear lamins with cytoskeletons, is referred to as the linker of nucleoskeleton and cytoskeleton (LINC) complex, and is involved in various nuclear events such as nuclear positioning and migration in somatic cells, as well as telomere motion and clustering in meiotic cells (Hiraoka and Dernburg 2009; Starr and Fridolfsson 2010).

During meiosis, the LINC complex‐mediated telomere motion promotes the pairing of homologous chromosomes (Hiraoka and Dernburg 2009; Lee et al. 2020b). Most organisms, including *S. pombe*, *C. elegans* , *A. thaliana* , and the mouse, use microtubules (MT) to drive the meiotic motion of the LINC complex; thus, motion and clustering of telomeres. In fission yeast, a SUN‐domain protein, Sad1 (Hagan and Yanagida 1995), forms a complex with a KASH‐domain protein, Kms1/2, on the NE (Miki et al. 2004). The Sad1‐Kms1/2 complex tethers kinetochores to the yeast MTOC, the Spindle Pole Body (SPB), in mitotic cells. Upon the induction of meiosis, Sad1‐Kms1/2 dissociates from kinetochores and binds to telomeres through the telomere‐binding protein complex, Bqt1‐Bqt2, and mediates the clustering of telomeres on the SPB (Chikashige et al. 2007). Kms1 interacts with dynein light chain, Dlc1, a component of dynein on cytoplasmic MTs (Miki et al. 2004). To promote chromosome pairing, the dynein‐driven force on the MT mediates oscillatory movement of the whole nucleus, with the SPB as the leading component. This movement of the yeast nuclei is called “horse‐tail” movement (Chikashige et al. 1994). In other organisms, cytoskeleton‐mediated motion of the LINC complex drives the motion of telomeres; thus, whole chromosomes inside of a nucleus, for the pairing of homologous chromosomes by increasing the chance of collision among the chromosomes and disrupting pairing of nonhomologous chromosomes.

As in the fission yeast, dynein‐mediated forces on MTs move the LINC complex in nematode and the mouse (Horn et al. 2013; Morimoto et al. 2012; Penkner et al. 2009; Sato et al. 2009; Yamamoto et al. 1999). In the nematode, the pairing center, a specific chromosome locus (MacQueen et al. 2005; Phillips et al. 2005), is moved by the LINC complex (Penkner et al. 2009). The LINC complex in *A. thaliana* meiosis uses meiosis‐specific kinesin for meiotic telomere motion (Cai et al. 2025).

Telomeres in the budding yeast, *Saccharomyces cerevisiae*, also cluster during meiotic prophase I. However, the clustering is unstable and less tight than in other organisms, called “loose” bouquet (Trelles‐Sticken et al. 1999). To drive telomere motion and clustering in meiotic cells, the yeast uses cytoplasmic actin filaments rather than MT (Koszul et al. 2008; Trelles‐Sticken et al. 2005). Actin filaments form cables with bundling proteins (Moseley and Goode 2006). The actin cables bind to a KASH‐domain protein, Mps2 (Fan et al. 2020; Lee et al. 2020a), which forms a complex with a meiosis‐specific ONM protein, Csm4 (Conrad et al. 2008; Kosaka et al. 2008; Wanat et al. 2008), through the interaction with Myo2, a yeast Type‐V myosin (Lee et al. 2020a). The SUN domain protein, Mps3, forms a complex with Mps2 on NE and binds to a meiosis‐specific telomere‐binding protein, Ndj1, to tether telomeres to INMs (Conrad et al. 1997; Rockmill and Roeder 1998).

Mps3 changes its localization on NE during meiosis (Bommi et al. 2019; Conrad et al. 1997; Prasada Rao et al. 2021). In mitotic cells, as a component of the half‐bridge near the SPB, Mps3 together with Mps2 forms a ring around the SPB (Chen et al. 2019). On the other hand, when yeast cells enter meiosis, Mps3 forms multiple ensembles on NEs in addition to one on the SPB. These Mps3 foci/patches with dynamic motion increase their number during meiotic Prophase I (Conrad et al. 2007; Prasada Rao et al. 2021). Upon the onset of meiosis I division, most of the Mps3 signals on NEs disappear, leaving two SPB‐associated spots (Conrad et al. 2007; Prasada Rao et al. 2021). The patch formation of the SUN‐domain proteins on NE during meiosis, in addition to MTOC, is also seen in fission yeast, Sad1, and nematodes, SUN‐1 (Chikashige et al. 2006; Sato et al. 2009; Yoshida et al. 2013).

Mps3 patches on meiotic NEs colocalize with clusters of NE‐bound Rap1, which binds telomeres and other genomic loci (Conrad et al. 2007). Actin‐driven force mediates the motion of Mps3 patches, and therefore telomeres, and, as a result, entire chromosomes (Conrad et al. 2008; Koszul et al. 2008; Trelles‐Sticken et al. 2005). The inhibition of actin cable formation in meiotic cells by a polymerization inhibitor, Latruncurin B (LatB), reduced Mps3 motion without affecting patch formation (Prasada Rao et al. 2021). Importantly, the formation of cytoplasmic actin cables is induced in meiotic Prophase I (Koszul et al. 2008; Takagi et al. 2021; Taxis et al. 2006). How actin cables/filaments are formed and control the Mps3‐Mps2‐Csm4 complex on NE in meiotic cells remains unsolved. Actin dynamics are mediated by various actin‐binding proteins in mitosis (Gonzalez Rodriguez et al. 2023; Moseley and Goode 2006). Yeast myosins, Myo1, Myo2, and Myo4, but not Myo5, bind to meiotic chromosome ends (Lee et al. 2020a). Meiosis‐specific expression of a dominant‐negative version of Type‐V myosin, Myo2, which binds to Mps2, reduced telomere motion (Lee et al. 2020a), suggesting the role of Myo2 in telomere dynamics during meiosis. Currently, actin bundles with Myo2 motor drive the motion and dissolve the clustering of Mps3 patches, thus, telomere clusters (Lee et al. 2020b). Alternatively, although not exclusively, actin polymerization per se facilitates meiotic telomere motion by a “piggy‐backing” mechanism (Koszul et al. 2008). In these models, the roles of most of the actin‐binding proteins in meiotic chromosome/telomere motion, including Mps3 dynamics, remain largely unknown. In addition, what kind of force controls the de‐clustering of Mps3 patches, which underlies loose telomere clustering, is unclear.

In this study, we re‐evaluated and confirmed the role of Myo2 in the motion of Mps3 patches on NEs by using meiosis‐specific depletion mutants. Myo2 is important for maintenance of telomere clustering in the absence of Rec8, a meiosis‐specific component of cohesin (Klein et al. 1999). We also showed a new role of Sac6 fimbrin, which cross‐links actin filaments in cables (Adams et al. 1991), in the formation of actin cables during meiosis. Our study indicates that Sac6‐mediated bundling/crosslinking of actin filaments is not important for Mps3 motion on NE, although actin polymerization is essential.

## 2 Results

### 2.1 Type‐V Myosin, Myo2, Is Critical for Mps3 Motion

In budding yeast, the formation of actin cables in the cytoplasm is induced during meiosis (Koszul et al. 2008; Taxis et al. 2006). Indeed, the treatment of meiotic cells with LatB reduced the motion of telomeres as well as unpaired or paired chromosomes (Koszul et al. 2008; Trelles‐Sticken et al. 2005). We confirmed the LatB reduced the motion of Mps3 ensembles during wild‐type meiosis (Conrad et al. 2007; Rao et al. 2011). Mps3, visualized as a GFP‐fusion protein, Mps3‐3xGFP (simply, Mps3‐GFP), changed its localization during meiotic Prophase I. At 0 h upon the induction of meiosis, it was predominantly localized to the SPB, as a single punctum (Figure 1A,B). After the induction of meiosis, Mps3 forms multiple foci or patches (hereafter patches) on the NE with heterogeneous motion. In early Prophase I, such as at 3 h, cells contained 3–5 patches (Figure 1C, Supplemental Figure 1). At the later time point of 5 h, the number of patches increased and covered most of the NEs (Figure 1C, Supplemental Figure 1). In some cells, a transient clustering of Mps3 patches is observed (e.g., 4 h in Figure 1A), which corresponds to a loose telomere bouquet (Trelles‐Sticken et al. 1999). Upon entering meiosis I (MI) division, most of the NE‐bound Mps3 signals disappear, leaving two Mps3 spots as a component of the SPB (e.g., 7 h in Figure 1A).

**FIGURE 1:**
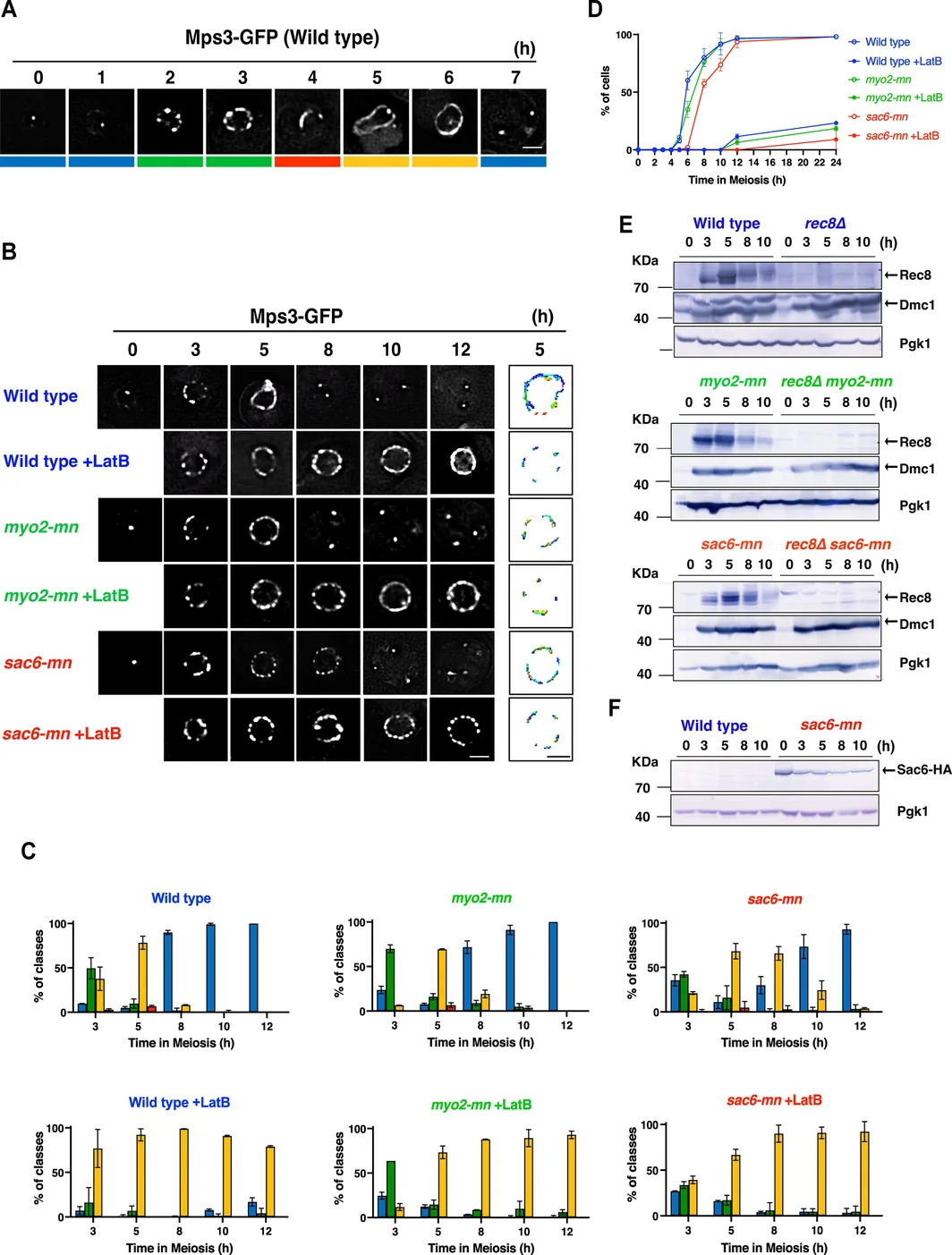
Myo2 promotes actin‐mediated Mps3 motion on NE. (A) Mps3‐GFP localization was analyzed in wild‐type cells (PRY64‐1/64‐2) at various time points during meiosis. Representative images at each time are shown. Based on Mps3‐GFP localization, cells with Mps3‐GFP were classified into four classes: single or double Mps3 focus (blue), 3–5 foci (green), multiple patches (orange), and clustering (red). Bar indicates 2 μm. (B) Time‐lapse analysis of Mps3‐GFP in various strains at different time points in meiosis. A single focal plane of a cell was analyzed, and representative images are shown on the left. The tracking analysis of Mps3 patches in various strains is shown on the right. Tracking (right panels) was performed for all Mps3 patches in a single representative cell for 30 s at 5 h in SPM. Each color shows the tracking of a single patch at a single focal plane. The inhibitor LatB (30 μM) was added at 3 h. Wild type, PRY64‐1/64‐2; *MYO2mn*, SPY137/139; *SAC6mn*, SPY142/143. Bar indicates 2 μm. (C) Based on Mps3‐GFP localization, cells with Mps3‐GFP were classified into four classes (A) and quantified: Single or double Mps3 focus (blue), 3–5 foci (green), multiple (> 5) patches (orange), and clustering (red). At each time point, more than 60 nuclei were counted. Means and SD (standard deviation) of three independent experiments are shown. (D) Meiosis I progression in different mutants. A nucleus was stained with DAPI, and the number of DAPI bodies in a cell was counted at each time point. Cells induced to undergo meiosis at 0 h were treated with LatB from 3 h. Means and S.D. of three independent experiments are shown. Wild type, blue circles; *MYO2mn*, green circles; *SAC6mn*, red circles: Closed symbols, cells treated with LatB from 3 h; open symbols, cells without LatB treatment. (E) Western blotting analysis of Rec8 and Dmc1 for the progression of prophase I. Samples at different time points were analyzed. The loading control was Pgk1. Wild type, PRY64‐1/64‐2; *MYO2mn*, SPY137/139; *SAC6mn*, SPY142/143; *rec8*, KYS271; *rec8 MYO2mn*, SPY145/146; *rec8 SAC6mn*, SPY150/151. (F) Western blotting analysis of HA‐Sac6. Samples at different time points were analyzed. The loading control was Pgk1. Wild type, PRY64‐1/64‐2; *SAC6mn*, SPY142/143.

Previous analyses of the movement of chromosome loci, including telomeres, in 2D and 3D spaces showed 3–4 components of their speed (Conrad et al. 2008; Nozaki et al. 2021). Since Mps3 patches are quite heterologous in their shape and number even in a single nucleus, we randomly picked up cells (up to 30 cells) and measured the track of a single patch in a cell for every 1 s for a short period of 30 s to simplify the analysis (Prasada Rao et al. 2021). An average speed of each patch in different nuclei in the population was calculated from continuous tracking over time. Each Mps3‐GFP patch moves at a speed of 0.01–0.25 μm/s in prophase I with an average speed of 0.09 μm/s (Figure 2A). When the cells were treated with LatB at 3 h, the localization pattern of Mps3 patches on NE was not largely affected (Figure 1B,C). On the other hand, the average speed decreased to 0.052 μm/s at 5 h (Figure 2A), confirming the actin polymerization‐dependent motion of Mps3 on NE. Note that velocities of Mps3 patches are much slower than those of chromosome loci reported previously (Conrad et al. 2008; Nozaki et al. 2021). This could be due to differences in the measurement methods and/or in the objects studied (Mps3 patches versus chromosome loci). To dissect the nature of the Mps3 motion, we applied a Gaussian mixture model to the Mps3 track in different time intervals (Figure 2B, top graphs) and found two components (light blue and red lines). Among the two components, the faster one (red lines in Figure 2B) is sensitive to the LatB treatment, while the slow component is insensitive (light blue lines, Figure 2B). This suggests that the motion of Mps3 patches is driven by actin (polymerization)‐dependent and ‐independent forces.

**FIGURE 2:**
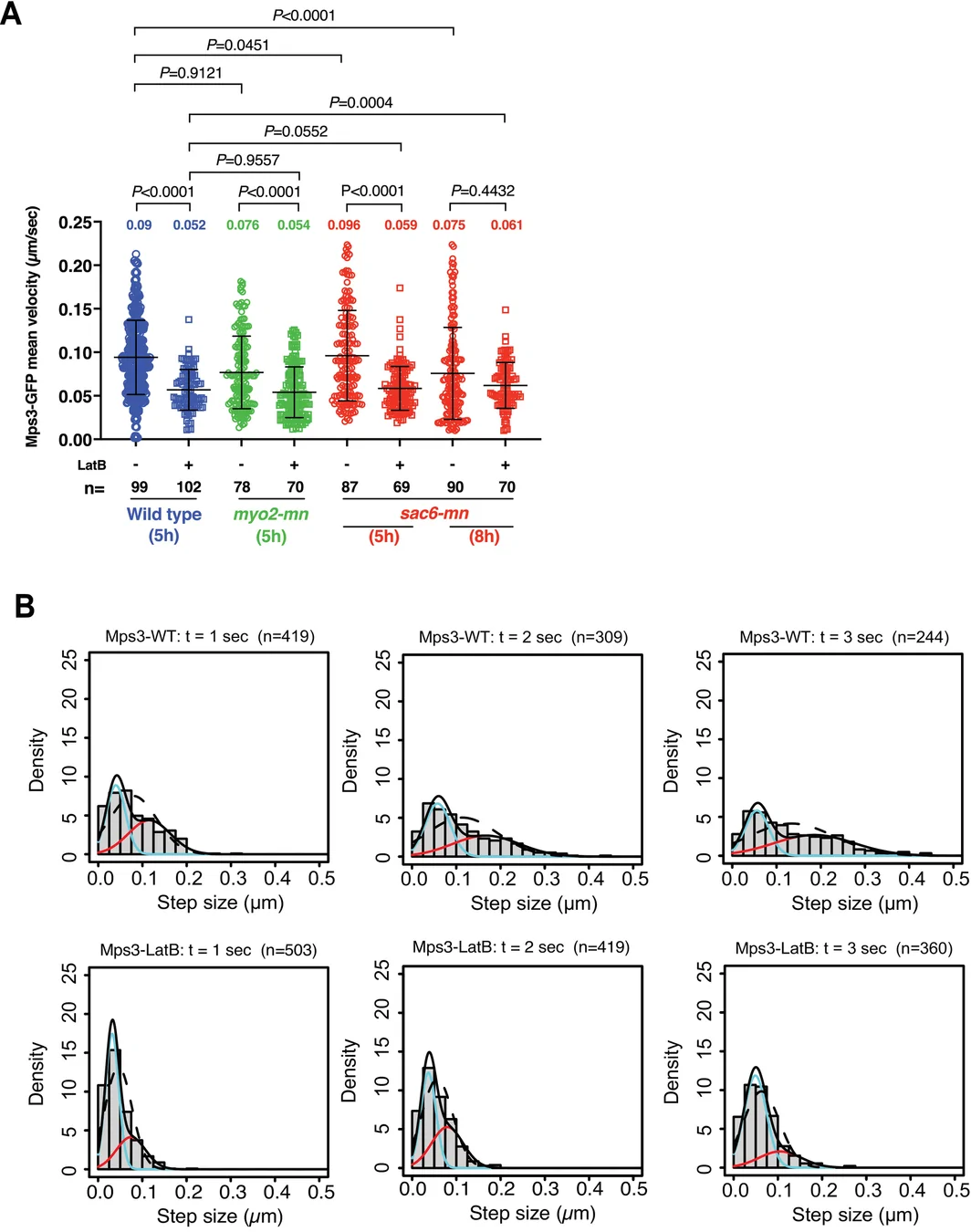
Myo2, but not Sac6, is necessary for meiotic Mps3 motion. (A) The velocity of Mps3 patches was measured in different strains. Time‐lapse images were taken every second for 30 s. Mps3 patches were identified and followed for their tracks. For each track, an average velocity was calculated based on the total length of the track of the patch. At each time point, 200 patches on randomly selected cells were analyzed. Means and SD are shown in black. Mean velocities and numbers of Mps3 patches are shown on the top and bottom, respectively. *p*‐Values were calculated and shown above. (B) The Gaussian mixture model was applied to the Mps3 track in different time intervals (1, 2, and 3 s). The histogram shows the density of each class of speeds (0.025 μm interval). The light blue (slower) and red (faster) lines indicate the two components of the fit. The black line and the dotted black line indicate the Gaussian mixture distribution and the single Gaussian distribution, respectively.

LatB treatment blocked cell entry into MI with persistent Mps3 patches on NE by 12 h (Figure 1C,D). A previous study showed that the treatment with the drug delayed the MI entry by ~2 h (Koszul et al. 2008). In our experiments, we used wild‐type cells with Mps3‐GFP, which delayed MI entry by ~2 h compared to wild‐type cells without the tag (Figure 1D) (Rao et al. 2011), suggesting a weak defect in meiotic prophase I. Mps3 tagging with GFP and LatB treatment exhibited a synergistic defect in MI entry. We need further analysis to dissect the prophase I defects conferred by the Mps3 tagging and/or the LatB treatment.

By expressing a dominant‐negative version of Type‐V myosin, Myo2, which is essential for mitotic growth, a previous study showed that Myo2 promotes telomere motion (Lee et al. 2020a). We extended the study by constructing a meiosis‐specific depletion mutant of the *MYO2* gene. For this, the endogenous *MYO2* promoter was replaced with the *CLB2* promoter, whose activity shuts down during meiosis but not during mitotic growth (Lee and Amon 2003). The *CLB2p‐MYO2*, hereafter *MYO2mn* (meiotic null), displayed normal onset of meiosis I (Figure 1D) with wild‐type spore viability (94.5% with 100 tetrads, compared with 96.0% in the control). Normal prophase I progression was also confirmed by western blotting analysis of two meiosis‐specific proteins, Rec8 and Dmc1 (Figure 1E). Like wild‐type control, *MYO2mn* cells formed Mps3 patches on NEs at 3 and 5 h with similar kinetics to wild‐type cells (Figure 1B,C). On the other hand, the average velocity of Mps3 patches in *MYO2mn* cells was 0.076 μm/s at 5 h, slower than in the control (Figure 2A, *p* = 0.0004, Mann–Whitney's *U*), confirming the role of Myo2 in Mps3 motion (Lee et al. 2020a). Importantly, the treatment of cells with LatB reduced the speed more than untreated *MYO2mn* cells (0.054 μm/s vs. 0.076 μm/s; *p* < 0.0001, Mann–Whitney's *U*; Figure 2A), supporting the idea that residual motion of Mps3 patches is dependent on actin polymerization. One possibility is that Myo2 depletion in the *MYO2mn* is not complete, leaving residual Myo2 activity.

To deplete Myo2 in meiotic cells more efficiently, we constructed cells with an auxin‐inducible degron (AID) tag (Nishimura et al. 2009) on Myo2, *MYO2‐AID*. The addition of auxin at 3 h after the induction of meiosis reduced Myo2 to very low levels (Figure 3A). Different from the *MYO2mn* mutant, Myo2 depletion by auxin‐dependent degradation induced arrest before entry into MI (Figure 3B). Western blots showed high‐level expression of Ndt80, a transcriptional activator for pachytene exit (Xu et al. 1995), and the appearance of phosphorylated Ndt80 as well as the expression of an Ndt80 downstream target, Cdc5 PLK kinase, even in the presence of auxin at 8 and 10 h (Figure 3A). This shows a meiotic arrest induced by Myo2 at late prophase (after the pachytene exit), suggesting a unique role of Myo2 in the entry into meiosis I. We confirmed that the meiosis‐specific localization pattern of Mps3 patches at 5 h was not affected by Myo2 depletion from 3 h (Figure 3C,D; Supplemental Figure 2). The Mps3 patches persisted at later time points, consistent with the Prophase I arrest induced by the depletion (Figure 3C,D). Myo2 deletion from 3 h reduced the velocity of Mps3 patches from 0.086 to 0.052 μm/s at 5 h (Figure 3E), which was similar to that in the presence of LatB (Figures 2A, 3E). This supports the idea that Myo2 promotes Mps3 motion together with actin polymerization. The Gaussian mixture model showed that the faster component, but not the slower component, is sensitive to Myo2 depletion (Figure 3F), supporting the idea that Myo2 moves Mps3 patches with actin polymerization.

**FIGURE 3:**
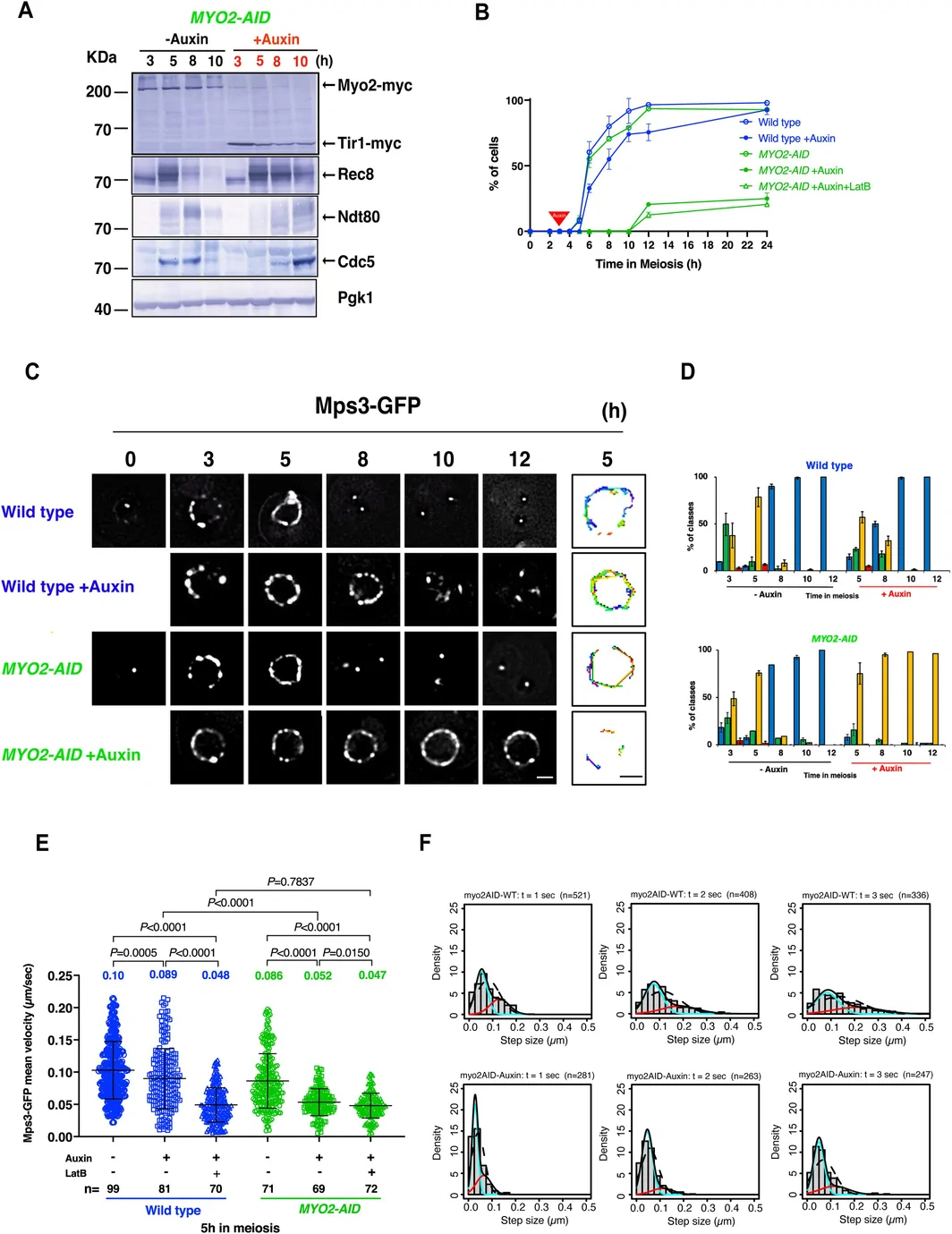
Myo2 is critical for Mps3 motion during meiosis. (A) Western blotting analysis of Myo2‐myc‐AID, Rec8, Ndt80, and Cdc5 for the progression of prophase I. Samples at different time points were analyzed. Copper (CuSO_4_, 25 mM) for Tir1 expression and Auxin (2 mM) was added at 2.5 and 3 h to a culture of *MYO2‐myc‐AID* cells (SPY321/322), respectively. The loading control was Pgk1. (B) Meiosis I progression in different mutants. A nucleus in a cell was stained with DAPI, and the number of DAPI bodies in a cell was counted at each time point. Cells induced to undergo meiosis at 0 h were treated with either auxin and/or LatB from 3 h. Mean and SD of three independent experiments are shown. Wild type, blue circles; *MYO2‐AID*, green circles: Closed symbols, cells treated with auxin from 3 h; open symbols, cells without auxin treatment. Green closed triangles, *MYO2‐AID* cells treated with auxin (2 mM) and LatB (30 μM) at 3 h. (C) Mps3‐GFP localization was analyzed in wild‐type (PRY64‐1/64‐2) and *MYO2‐myc‐AID* cells (SPY321/322) at various time points during meiosis. Auxin (2 mM) was added at 3 h. Representative images at each time point are shown. The images were taken and processed as shown in Figure 1. Tracing (right panels) was performed for all Mps3 patches in a single representative cell for 30 s at 5 h. Bar indicates 2 μm. (D) Based on Mps3‐GFP localization, cells with Mps3‐GFP were classified into four classes (A) and quantified: Single or double Mps3 focus (blue), 3–5 foci (green), > 5 patches (orange), and clustering (red). At each time point, more than 60 nuclei were counted. Mean and SD of three independent experiments are shown. (E) The velocity of Mps3 patches was measured in different strains. Time‐lapse images were taken every second for 30 s. Mps3 patches were identified and followed for their tracks. For each track, an average velocity was calculated based on the length of the total track of the patch. At each time point, 200 patches (a number of patches) were analyzed. Mean and SD are shown in black. Mean velocities and numbers of Mps3 patches are shown on the top and the bottom, respectively. *p*‐Values were calculated and shown above. (F) The Gaussian mixture model was applied to the Mps3 track in different time intervals (1, 2, and 3 s). The histogram shows the density of each class of speeds (0.025 μm interval). The light blue (slower) and red (faster) lines indicate the two components of the fit. The black line and the dotted black line indicate the Gaussian mixture distribution and the single Gaussian distribution, respectively.

To confirm the role of Myo2 in the establishment of Mps3 patches, we depleted Myo2 at 2 h after the induction of meiosis, which is just before the formation of Mps3 patches on NE. The Myo2 depletion did not affect the appearance of Mps3 patches (Supplementary Figure 3), which is consistent with the result that early treatment of meiotic cells with LatB did not affect the formation of Mps3 patches (Rao et al. 2011).

Note that the treatment of wild‐type cells with auxin delayed the onset of meiosis I by ~1 h (Figure 3B). Moreover, the presence of auxin in wild‐type cells significantly reduced Mps3 velocity from 0.1 to 0.089 μm/s (*p* = 0.0005, Figure 3E). These indicate the careful interpretation of the meiotic phenotypes by the AID‐mediated protein depletion.

We also examined cytoplasmic localization of actin polymers, including the cables induced during meiosis (Taxis et al. 2006). Actin polymers were visualized by Phalloidin staining (Figure 4A). In the wild‐type, cortical actin patches were observed predominantly at 0 h (before meiosis induction). ~25% of cells also contained cables (Adams et al. 1991). After the induction of meiosis, as shown previously (Koszul et al. 2008), in addition to cortical patches, multiple actin cables were induced in the cytoplasm during the meiotic Prophase I (3 and 5 h) (Figure 4A,B). At 5 h, ~75% of cells are positive for actin cables. In some cases, we observed internal actin patches together with cables (Figure 4A). At 10 h, most of the actin cables disappeared while cortical actin patches persisted even after meiosis I and II, as shown previously (Suda et al. 2007). When cells were treated with LatB at 3 h, actin cables and internal patches disappeared very quickly, even upon treatment (Figure 4A,B). In contrast, cortical actin patches are resistant to the LatB treatment. When the *MYO2mn* cells were stained with Phalloidin, we observed the induction of actin cables and internal patches in the mutant at 3 and 5 h, which were sensitive to the LatB treatment (Figure 4A,B). This suggests that the formation of actin cables is independent of Myo2 (or other Type‐V myosin, such as Myo5, may have a redundant function with Myo2). We also confirmed that Myo2 depletion in *MYO2‐AID* cells with auxin treatment did not affect actin cable formation in meiotic cells by phalloidin staining (Figure 4). The normal actin cable formation in the *MYO2mn* mutant was confirmed by examining the localization of actin‐filament binding protein, Abp140 (see below, Figure 5).

**FIGURE 4:**
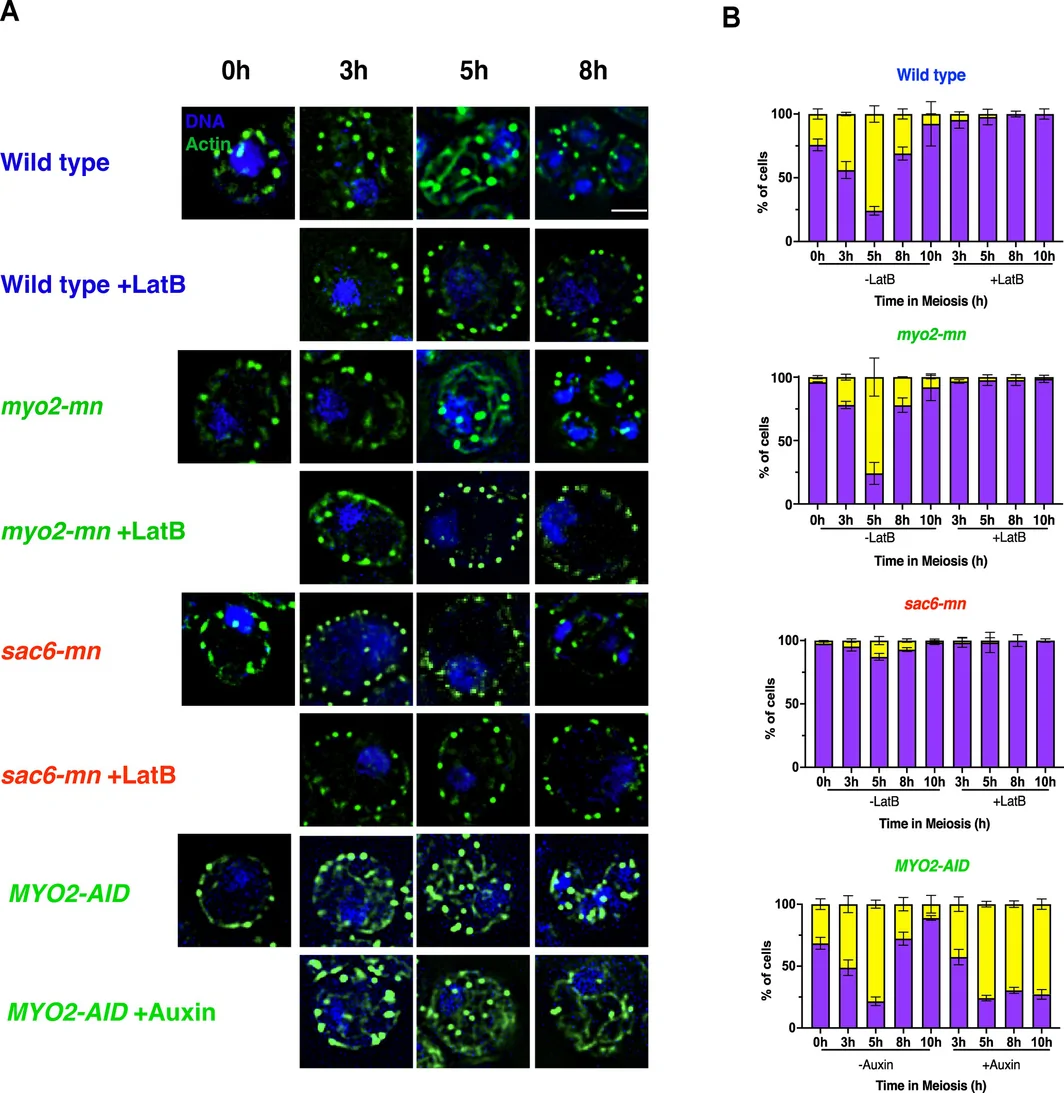
Sac6 promotes actin cable formation during meiosis. (A) Actin polymers were visualized in wild‐type cells (PRY64) at various time points during meiosis by staining of whole cells with Phalloidin (green) and DAPI (light blue). Representative images at each time are shown. Wild type, PRY64‐1/64‐2; *MYO2mn*, SPY137/139; *SAC6mn*, SPY142/143; *MYO2‐myc‐AID*, SPY321/322. The bar indicates 2 μm. (B) Phalloidin staining patterns were classified into two classes: cortical actin patches (purple), cortical actin patches and actin cables (yellow). At each time point, more than 100 nuclei were counted. Means and SD of three independent experiments are shown.

**FIGURE 5:**
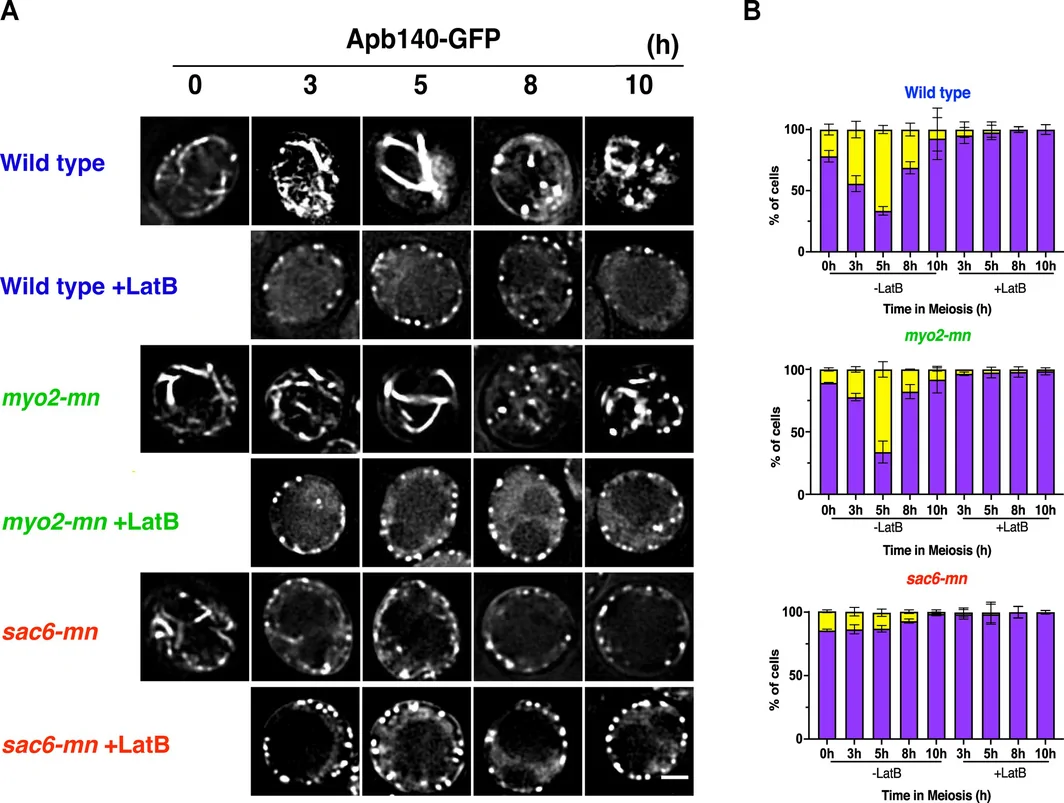
Abp140 cable formation is reduced upon Sac6 depletion. (A) Abp140‐GPF was visualized in wild‐type and mutant cells at various time points during meiosis. Representative images at each time are shown. Wild type, HYG543/544; *MYO2mn*, SPY323/324; *SAC6mn*, SPY209/210. Bar indicates 2 μm. (B) Staining patterns of Abp140 signals were classified into two classes: cortical actin patches (purple) and cortical actin patches and actin cables (yellow). At each time point, more than 100 nuclei were counted. Means and SD of three independent experiments are shown.

### 2.2 Sac6 Fimbrin Is Critical for the Formation of Actin Cables but Not for Mps3 Motion

The *SAC6* gene encodes a yeast fimbrin, which bundles actin filaments for actin cables (Adams et al. 1991). Sac6 is critical for endocytosis (Hill et al. 2024) and the maintenance of cell polarity (Adams et al. 1991). The *sac6* deletion mutant is temperature‐sensitive for mitotic growth (Adams et al. 1991). To avoid the effect of mitotic defects on meiotic phenotypes, we also constructed a meiotic null mutant of *SAC6*, *CLB2p‐HA‐SAC6* (*SAC6mn*). The level of the HA‐Sac6 protein at 5 h decreased to ~1/3 relative to that at 0 h (Figure 1F). The *SAC6mn* mutant delayed the entry into meiosis I by ~2 h, suggesting a defect in meiotic Prophase I (Figure 1D). The *SAC6mn* mutant reduced spore viability to 85.0% (100 tetrads), indicating an important role in meiosis.

First, we checked the formation of actin cables in the *SAC6mn* mutant by phalloidin staining. As shown above, about 40% and 75% of wild‐type cells are positive for actin cables at 3 and 5 h in meiosis, respectively (Figure 4A,B). *SAC6mn* mutant cells largely decreased the formation of actin cables. Only ~10% of cells contained actin cables at 5 h. This suggests that Sac6 is critical for the formation of actin cables in meiotic cells, as shown for mitotic actin cables in the *sac6* null mutant (Adams et al. 1991). To confirm the decreased actin cables upon *SAC6* depletion, we also examined the localization of a GFP fusion of Abp140 (Abp140‐4xGFP), which binds to actin filaments as well as patches and is often used as a marker for actin cables (Asakura et al. 1998; Yang and Pon 2002). As reported previously (Koszul et al. 2008; Taxis et al. 2006), the formation of Abp140‐4xGFP cables was induced during meiosis, such as 3 and 5 h, in wild‐type control cells (Figure 5). The cable was sensitive to the LatB treatment (Figure 5). The treatment left the cortical patch. The *MYO2mn* mutant is proficient in Abp140‐cable formation. Importantly, in the *SAC6mn* mutant, Abp140‐GFP cables were largely reduced during meiosis, while cortical actin patches were dominant in the depletion mutant (Figure 5A,B). Residual Abp140‐cable staining in the *SAC6mn* mutant was abolished with the LatB treatment. These results support the idea that actin cable formation in meiotic cells depends on Sac6 fimbrin.

To know the role of the Sac6 fimbrin in Mps3 dynamics during meiosis, we examined the localization of Mps3‐GFP in the *SAC6mn* mutant cells. The Mps3 patches appeared from 3 h, as in the wild type (Figure 1B,C). At 5 h, more cells are positive for Mps3 patches covering the NE. Due to the delay in prophase I, more than ~60% of *SAC6mn* cells still showed the patches at 8 h. At 10 h, most of the Mps3 on NEs disappeared, with 2–4 spots (Figure 1C). This showed the normal Mps3 patch formation in the *SAC6*‐depleted cells.

When the velocity of Mps3 patches at 5 h in the mutant was measured, the average velocity was 0.096 μm/s, which is indistinguishable from that in the wild‐type control (versus the velocity at 5 h in wild type; *p* = 0.05, Mann–Whitney's *U*; Figure 2A). At 8 h, the velocity was 0.075 μm/s. Importantly, the treatment of the meiotic *SAC6mn* cells with LatB at 3 h decreased the velocity to 0.059 μm/s at 5 h (compared to that without the treatment, *p* = 0.014, Mann–Whitney's *U*), indicating the presence of actin polymerization‐mediated motion of the Mps3 patches in the *SAC6mn* mutant. This suggests that Sac6, therefore, Sac6‐mediated actin filament bundling is not critical for the motion of Mps3 on the NE.

### 2.3 Myo2 Is Necessary for the Maintenance of Mps3 Clustering in the *rec8* Mutant

To confirm the role of Myo2 and Sac6 in actin‐mediated Mps3 dynamics on NEs, we used the *rec8* mutant, which is deficient in sister chromatid cohesin in meiotic cells (Klein et al. 1999). Interestingly, the *rec8* mutant is known to cluster Mps3 patches as well as telomeres in one area on NE without resolution (Bommi et al. 2019; Trelles‐Sticken et al. 2005). In the *rec8* mutant, the clustering began at 3 h and persisted during further incubation (Figure 6A,B). Since the *rec8* mutant showed severe delay in the progression of meiosis I (Figure 6C), as shown (Klein et al. 1999), even at 12 h, most of the mutant cells were positive for NE‐bound Mps3 signals. The disruption of actin polymerization with LatB quickly resolved the clustering of Mps3 patches on NE (Bommi et al. 2019) (Figure 6A,B). This suggests that Mps3 clustering is a sensitive readout for the actin polymerization‐mediated force for Mps3 motion.

**FIGURE 6:**
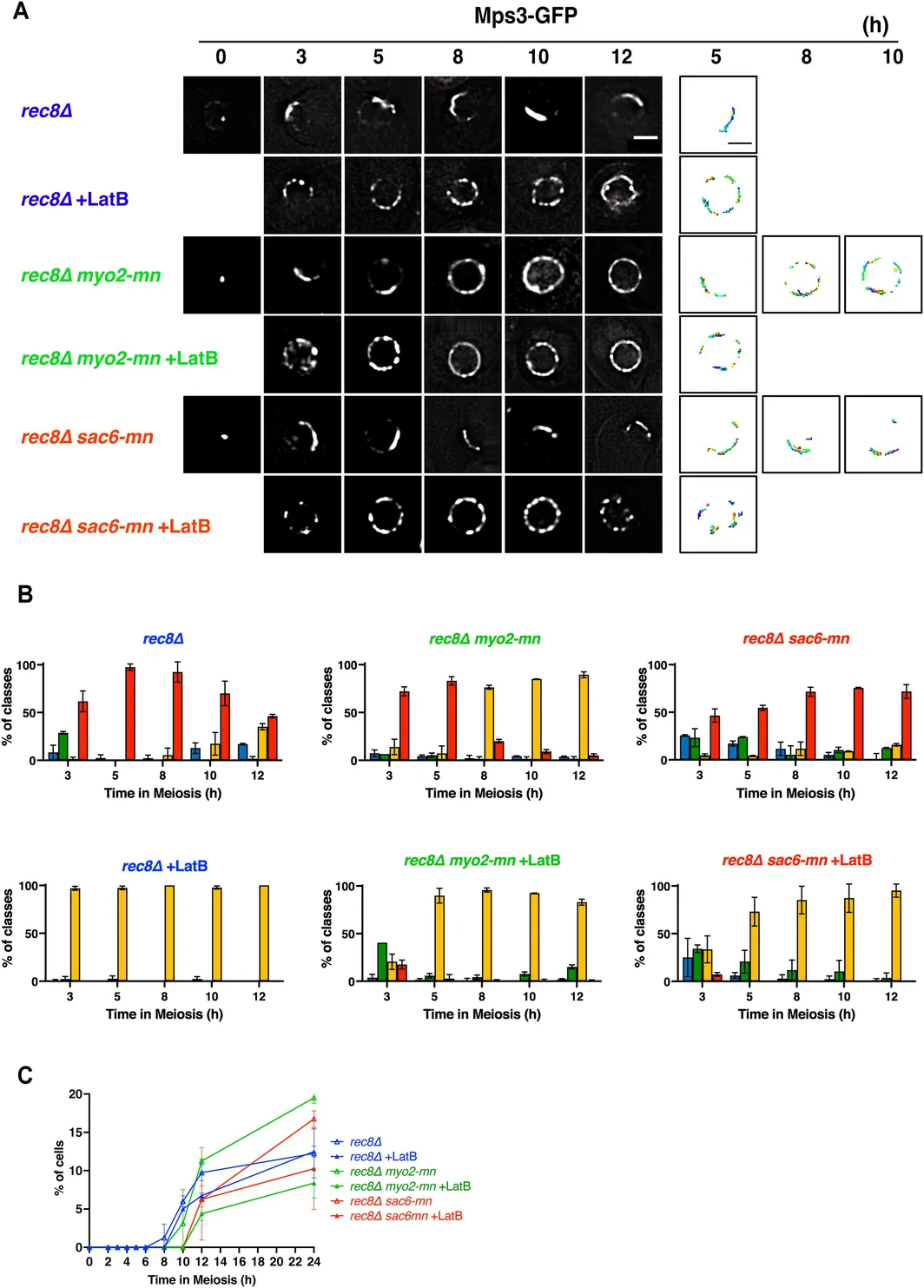
Mps3 localization in the absence of Rec8. (A) Mps3‐GFP localization was analyzed in *rec8* mutant cells (KYS271) at various time points during meiosis. Representative images at each time are shown on the left. The tracking analysis of Mps3 patches in various strains was shown on the right. Tracking was performed for all Mps3 patches in a single representative cell for 30 s at 5 h in SPM. Each color shows the tracking of a single patch at a single focal plane. *rec8*, KYS271; *rec8 MYO2mn*, SPY145/146; *rec8 SAC6mn*, SPY150/151. Bar indicates 2 μm. (B) Based on Mps3‐GFP localization, cells with Mps3‐GFP were classified into four classes (A) and quantified: Single Mps3 focus (blue), 2–5 foci (green), multiple patches (orange), and clustering (red). At each time point, more than 100 nuclei were counted. Means and SD of three independent experiments are shown. (C) Meiosis I progression in different mutants. A nucleus was stained with DAPI, and the number of DAPI bodies in a cell was counted at each time point. Cells induced to undergo meiosis at 0 h were treated with LatB (30 μM) from 3 h. Mean and SD of three independent experiments are shown. *rec8*, KYS271, blue tringles; *rec8 MYO2mn*, SPY145/146, red triangle; *rec8 SAC6mn*, SPY150/151, green triangle. Open symbols are for cells in the absence of LatB, while Closed symbols are for cells treated with LatB from 3 h.

First, we studied the role of Myo2 in Mps3 clustering in the *rec8* mutant. The *rec8 MYO2mn* showed Mps3 clustering at 3 and 5 h, as seen in the *rec8* mutant (Figure 6A,B). However, at 8 h (or later), *rec8 MYO2mn* resolved the clustering with NE‐covered Mps3 patches. Importantly, the addition of LatB at 3 h resolved the clustering quickly. This suggests the role of Myo2 in the maintenance of Mps3 clustering in the absence of Rec8. The absence of Mps3‐cluster resolution at early time points in the mutant could be due to partial depletion of Myo2 in the *MYO2mn* cells.

We also checked the effect of Sac6 depletion on the resolution of Mps3 clusters in the *rec8* mutant (Figure 6A,B). The *rec8 SAC6mn* cells accumulated Mps3 clusters during prophase I, as seen in the *rec8* mutant. The treatment of the *rec8 SAC6ms* cells with LatB resolved the Mps3 clustering. This supports our conclusion that Sac6, probably the actin cable, is not important for actin‐mediated motion of Mps3 patches on the NE and the maintenance of the clustering.

Finally, we checked the actin cables in the *rec8* mutant (Figure 7A). To our surprise, the *rec8* cells form a novel structure of actin polymerization. As in wild type (Figures 4 and 5), the *rec8* mutant contains internal actin cables (Figure 7A). However, in addition, the actin cables with cortical patches appeared near the plasma membrane and/or cell wall, thus forming cortical actin cables (Figure 7A,B), which were rare in the wild‐type cells. On the other hand, most of the *rec8* mutant cells contained the cortical cables at late time points (Figure 7C). The cortical actin cables were sensitive to treatment with LatB, but their formation is independent of Myo2 and Sac6 (Figure 7A,C). Sac6‐independent cortical cables are different from internal actin cables, which depend on Sac6 (Figures 4 and 5). One possibility is that cortical actin patches are connected to form a cable‐like structure below the membrane. We need further work to dissect the nature of these unique actin cables.

**FIGURE 7:**
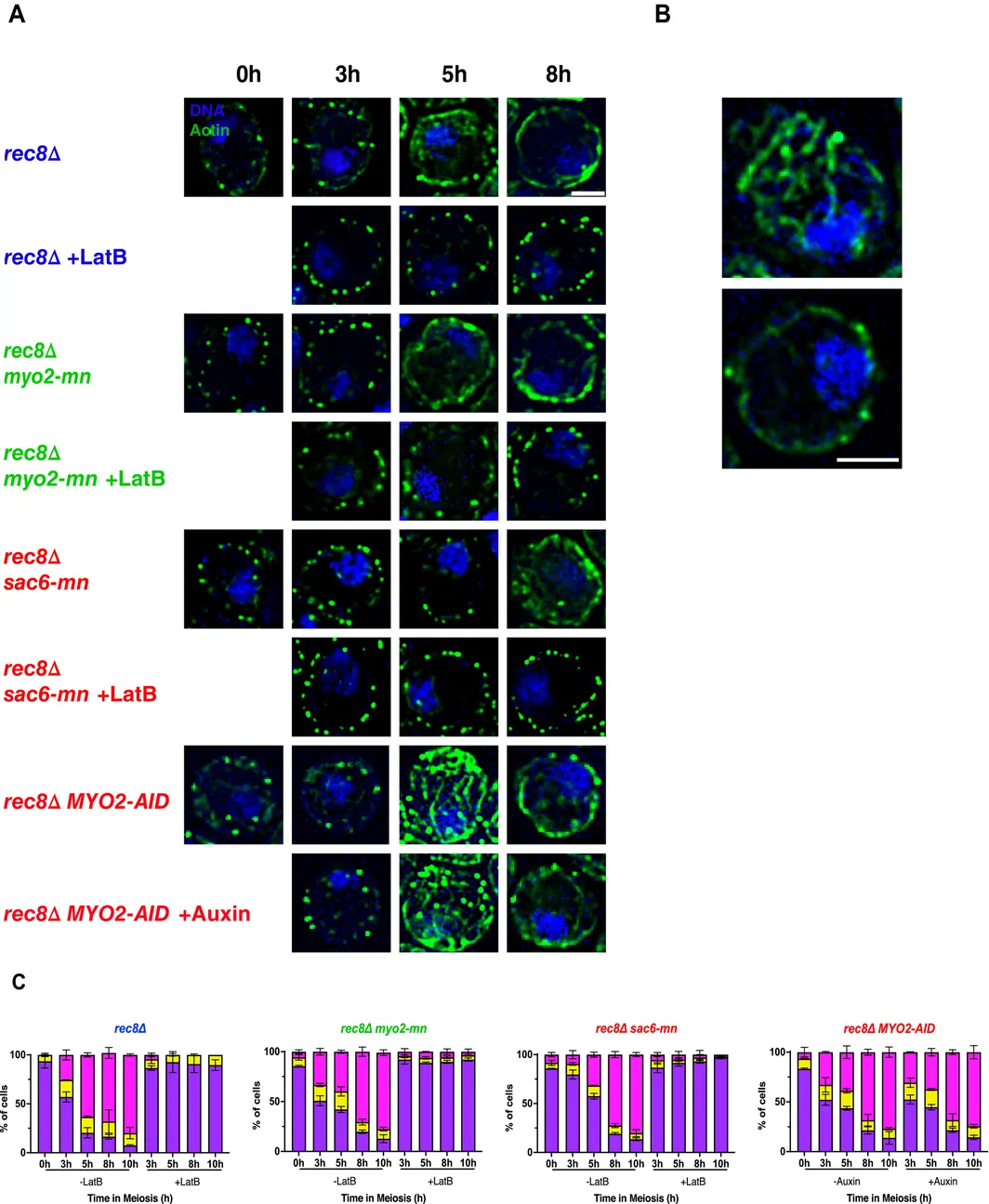
Actin cable formation in the absence of Rec8. (A) Actin polymers were visualized in wild‐type cells (PRY64) at various time points during meiosis by staining of whole cells with Phalloidin (green) and DAPI (light blue). Representative images at each time point are shown. *rec8*, KYS271; *rec8 MYO2mn*, SPY145/146; *rec8 SAC6mn*, SPY150/151; *rec8 MYO2‐AID*, SPY325/326. The bar indicates 2 μm. (B) Representative image of internal (top) and cortical (bottom) actin cables in the *rec8* mutant. The bar indicates 2 μm. (C) Phalloidin staining patterns were classified into three classes: Internal cables and cortical patches with cortical actin patches (magenta), cortical actin patches and internal actin cables without cortical cables (yellow), and cortical actin patches alone (purple). At each time point, more than 100 nuclei were counted. Means and SD of three independent experiments are shown.

## 3 Discussion

The cytoskeletons move the LINC complex on the NE during meiosis to facilitate telomere motion, which is associated with the clustering of telomeres. Most organisms, including fission yeast, *C. elegans, Arabidopsis*, and mouse, utilize microtubules for the meiotic motion of the LINC complex (Cai et al. 2025; Chikashige et al. 2006; Morimoto et al. 2012; Sato et al. 2009). In microtubule‐based motion, dynein‐ or kinesin‐mediated forces on microtubules move the LINC complex. On the other hand, the budding yeast utilizes actin polymerization, likely actin filaments or cables, for the meiotic motion of the LINC complex. In the cytoplasm of the yeast, actin forms cables as well as cortical patches (Moseley and Goode 2006). The formation of internal actin cables is induced during meiotic prophase I (Koszul et al. 2008; Taxis et al. 2006). In mitotic cells, various actin‐binding proteins, including myosin, control the dynamics of actin cables and patches (Gonzalez Rodriguez et al. 2023; Moseley and Goode 2006). However, little is known about how actin‐binding proteins regulate the motion of a budding yeast LINC complex in meiotic cells. A recent study showed the role of Type‐V myosin, Myo2, in the motion of the *S. cerevisiae* LINC complex, Mps3‐Mps2‐Csm4 (Lee et al. 2020a), supporting that myosin‐V‐mediated forces on actin filaments drive the motion of the LINC complex. However, the role of other actin‐binding proteins in the motion of the yeast LINC complex has not been addressed in detail. In this study, we confirmed the role of Myo2 in Mps3 motion by analyzing a meiosis‐specific depletion mutant of *MYO2*. Importantly, since Myo2 depletion resolved Mps3 clustering in the *rec8* mutant, it is likely that Myo2 plays a critical role in the maintenance of telomere clustering. Since wild‐type cells form the clustering transiently (Conrad et al. 2007; Trelles‐Sticken et al. 1999), at this point, it is not clear whether Myo2‐mediated force resolves the loose clusters of Mps3 or telomeres in wild type.

In addition, we studied the role of yeast fimbrin, Sac6, an actin filament‐bundling protein, in Mps3 dynamics. Indeed, the *SAC6* depletion largely reduced the cytoplasmic actin cables in meiotic cells. However, Sac6 depletion did not affect Mps3 motion. It is likely that the Mps3 motion does not depend on bundling/crosslinking of actin filaments or actin cables but does depend on actin polymerization. A simple interpretation is that a single or a few actin filaments, which could not be visualized with Phalloidin staining and Abp140‐GFP, are likely to play a role in the motion. However, at this point, we cannot deny the possibility that “invisible” actin cables, which are insensitive to the detection methods used in this study, play a role in the Mps3 motion. Given that another actin‐crosslinking protein, Scp1, plays a redundant role with Sac6 in endocytosis (Goodman et al. 2003; Hill et al. 2024), further investigation of various actin‐binding proteins is necessary to determine the relationship between the actin cable and/or filament and the motion of the LINC complex during meiosis. Moreover, several questions about the role of actin filament dynamics in budding yeast meiosis remain unsolved. Particularly, how are actin cables induced specifically during meiotic prophase I? Where does the nucleation of actin filaments occur in meiotic cells? How do actin filaments mediate telomere clustering without the organizing center? These questions should be addressed in future studies.

## 4 Experimental Procedures

### 4.1 Strains

All strains described here are derivatives of SK1 diploids, MSY832/833 (*MATα/MAT**a**, lys2/lys2, ura3/ura3, leu2::hisG/leu2::hisG*). ABP140‐4xGFP (Taxis et al. 2006) was obtained from Dr. M. Knopp. The genotypes of each strain used in this study are described in Table S1. *MPS3‐GFP* diploid cells showed wild‐type spore viability, but both showed ~2 h delay in the entry of meiosis I (Rao et al. 2011).

### 4.2 Strain Construction

The *CLB2p‐MYO2* and *CLB2p‐SAC6* genes were constructed by one‐step DNA integration using a PCR‐amplified DNA fragment with a template plasmid with the *KamMX‐pCLB2* cassette and primers shown in Table S2. The *MYO2‐myc‐AID* genes were constructed by one‐step DNA integration using a PCR‐amplified DNA fragment with a template plasmid with the *Myc‐AID‐Hph* cassette and primers shown in Table S2. DNA changes were confirmed by DNA sequencing of PCR‐amplified products.

### 4.3 Anti‐Serum and Antibodies

Anti‐c‐Myc (Nakarai Tesque, MC045), Anti‐HA (Covance, 16B12), Anti‐Pgk1 (Abcam, 22C5D8), rabbit anti‐Rec8 (Zhu et al. 2011), and rabbit anti‐Dmc1 (Sasanuma et al. 2013) were used for Western blotting. The second antibody for Western blotting was an alkaline phosphatase‐conjugated secondary antibody (Promega).

### 4.4 Meiotic Time Course

*Saccharomyces cerevisiae* SK1 strains were patched onto YPG plates (2% bacteriological peptone, 1% yeast extract, 3% glycerol, 2% bacteriological agar) and incubated at 30°C for 12 h. Cells were inoculated into YPD plates (2% bacteriological peptone, 1% yeast extract, 2% glucose, 2% bacteriological agar) and grown for 2 days to obtain isolated colonies. A single colony was inoculated into YPD liquid medium and grown to saturation at 30°C overnight. To synchronize cell cultures, the overnight cultures were transferred to a pre‐warmed SPS medium (1% potassium acetate, 1% bacteriological peptone, 0.5% yeast extract, 0.17% yeast nitrogen base with ammonium sulfate and without amino acids, 0.5% ammonium sulfate, 0.05 M potassium bi‐phthalate) and grown for 17–18 h. Meiosis was induced by transferring the SPS‐cultured cells to pre‐warmed SPM medium (1% potassium acetate, 0.02% raffinose). The SPM‐cultured cells were harvested at various times after being transferred to the SPM medium. For auxin‐mediated depletion, 25 mM CuSO_4_ and 2 mM auxin were added at 2.5 and 3 h, respectively.

### 4.5 Cytology

Time‐lapse images of Mps3‐GFP and Abp140‐GFP were captured using a computer‐assisted fluorescence microscope system (DeltaVision; Applied Precision). The objective lens was an oil immersion lens (100×; NA, 1.35). Image deconvolution was performed using an image workstation (SoftWorks; Applied Precision). Time‐lapse image acquisition was performed every 0.5–1 s at a single focal plane. Meiotic cell cultures (~150 μL) were placed on bottom‐glass dishes (Matsunami) pre‐coated with Concanavalin A (10 mg/mL, Sigma).

Tracking of Mps3‐GFP was analyzed using the ImageJ program (NIH) and plug‐in MTrack2 (Nico Shuuman). Tracking and velocity analysis of Mps3‐GFP were analyzed using Imaris software (Oxford Instrument). 80 nuclei were counted.

The multi‐component Gaussian mixture model is fitted to the step‐size data of the Mps3 patches generated by Imaris, using the mixtools R package (Benaglia et al. 2009).

Latrunculin B (Calbiochem; cast‐428,020) was dissolved in DMSO at a concentration of 10 mM and used at 30 μM.

For visualization of actin polymers in yeast cells, the cells were fixed with 37% (V/V) formaldehyde and stained with Phalloidin (Invitrogen, A12381) according to the manufacturer's instructions.

### 4.6 Western Blotting

Western blotting was performed as described previously (Shinohara et al. 2000), using cell lysates extracted by the TCA method. After being harvested and washed twice with 20% TCA, cells were roughly disrupted with zirconia beads by the Multi‐beads shocker (Yasui Kikai). The protein precipitate recovered by centrifugation was suspended in SDS‐PAGE sample buffer adjusted to pH 8.8 and then incubated at 95°C for 10 min. After electrophoresis, the proteins were transferred onto a nylon membrane (Immobilon‐P, Millipore) and incubated with primary antibodies in a blocking buffer (1× PBS, 0.5% BSA) and then with alkaline phosphatase‐conjugated secondary antibody (Promega). The color reaction was developed with NBT/BCIP solution (Nacalai Tesque).

## Author Contributions

**Shuangping Cheng:** investigation, validation, methodology, formal analysis, data curation. **Tadasu Nozaki:** software, formal analysis, validation, writing – review and editing, investigation, visualization. **Yurika Fujita:** methodology, data curation, validation, writing – review and editing, visualization. **Masaru Ito:** writing – review and editing, methodology, data curation. **Akira Shinohara:** conceptualization, validation, supervision, project administration, writing – review and editing, writing – original draft, visualization, funding acquisition.

## Funding

This work was supported by the Institute for Fermentation, Osaka, to A.S.

## Ethics Statement

The authors have nothing to report.

## Consent

All authors have reviewed the manuscript and consented to its publication.

## Conflicts of Interest

The authors declare no conflicts of interest.

## Supporting information


**Table S1:** Strain list.
**Table S2:** Oligonucleotide list.
**Supplementary Figure 1**. Representative images of cells with Mps3‐GFP at 3 and 5 h are shown. The LatB (30 μM) was added at 3 h. Wild type, PRY64‐1/64‐2; *MYO2mn*, SPY137/139; *SAC6mn*, SPY142/143. Bar indicates 10 μm.
**Supplementary Figure 2**. Representative images of cells with Mps3‐GFP at 3 and 5 h are shown. The auxin was added at 3 h. Wild type, PRY64‐1/64‐2; *MYO2‐AID*, SPY321/322; *rec8 MYO2‐AID*, SPY325/326. The bar indicates 10 μm.
**Supplementary Figure 3**. Mps3‐GFP localization was analyzed in wild‐type (PRY64‐1/64‐2) and *MYO2 myc‐AID* cells (SPY321/322) at various time points during meiosis. Auxin (2 mM) was added at 2 h. Representative images at each time point are shown. The images were taken and processed as shown in Figure 1.

## Data Availability

The genotypes of strains and sequences of primers used for the strain construction are indicated in Supporting Information Tables. The raw data of images and materials are available upon request.
