# Supplementary material for: The Role of Actin‐Filament Bundling Protein, Fimbrin Sac6, and Type‐V Myosin Myo2, in the Motion of the SUN‐Domain Protein, Mps3, During Yeast Meiosis
Source: Genes Cells. 2026 Oct 28;31(6):e70156. doi: 10.1111/gtc.70156 (PMC13716544; doi:10.1111/gtc.70156)
Supplement: Supplementary file 1 [file GTC-31-0-s001.pdf]

Supplementary Information

Table S1. Strain List

Strain number	Genotypes
MSY832	*MAT**a**, ho::LYS2, lys2, ura3, leu2::hisG, trp1::hisG*
MSY833	*MATα, ho::LYS2, lys2, ura3, leu2::hisG, trp1::hisG*
PRY64-1/64-2	*MAT**a**/α, ho::LYS2/ho::LYS2, lys2/lys2, ura3/ura3, leu2::hisG/leu2::hisG, leu2::hisG/leu2::hisG, trp1::hisG/ trp1::hisG, MPS3-3xGFP::KanMX/MPS3-3x GFP::KanMX*
KYS271	PRY64-1/64-2 with *rec8Δ::KanMX/rec8Δ::KanMX*
SPY137/139	PRY64-1/64-2 with *pCLB2-MYO2::KanMX/pCLB2-MYO2:: KanMX*
SPY142/143	PRY64-1/64-2 with *pCLB2-SAC6::KanMX/pCLB2-SAC6::KanMX*
SPY145/146	PRY64-1/64-2 with *pCLB2-MYO2::KanMX/pCLB2-MYO2:: KanMX*, *rec8Δ::KanMX/rec8Δ::KanMX*
SPY150/151	PRY64-1/64-2 with *pCLB2-SAC6::KanMX/pCLB2-SAC6::KanMX, rec8Δ::KanMX/rec8Δ::KanMX*
KYS303/304	*MAT**a**/α, ho::LYS2/ho::LYS2, lys2/lys2, ura3/ura3, MPS3-FLAG::KanMX/MPS3-FLAG::KanMX, NDJ1-HA:: KanMX/NDJ1-HA:: KanMX*
SPY321/322	PRY64-1/64-2 with *MYO2-AID-myc::HphMX/MYO2-AID-myc::HphMX, TIR1-myc::URA3/TIR1-myc::URA3*
HYG543/544	*MAT**a**/α, ho::LYS2/ho::LYS2, lys2/lys2, ura3/ura3, ABP140-4xGFP::KanMX/ABP140-4xGFP::KanMX*
SPY209/210	HYG543/544 with *pCLB2-SAC6::KanMX/pCLB2-SAC6::KanMX*
SPY323/324	HYG543/544 with *pCLB2-MYO2::KanMX/pCLB2-MYO2:: KanMX*
SPY325/326	PRY64-1/64-2 with *MYO2-AID-myc::HphMX/MYO2-AID-myc::HphMX, TIR1-myc::URA3/TIR1-myc::URA3, rec8Δ::KanMX/rec8Δ::KanMX*

Table S2. Oligonucleotide list

Primer name	Sequence
Clb2Myo2-F	5'-TTTTGTTTTCTAAGGTACTATTCGACACCATTCCATTGGACAGCGATACTGAATTCGAGCTCGTTTAAAC
Clb2Myo2-R	5'-CCCAATTCTTTATGGGGATACCAGCATCGTGTACCCACTTCAAAAGACATGCACTGAGCAGCGTAATCTG
Clb2Sac6-F	5'-TTATAATGAAGATTTATCGATATAAATTTTGGTTATTTCAGGAGAACAAGGAATTCGAGCTCGTTTAAAC
Clb2Sac6-R	5'-GTGTGGGAATTCGAGAAAGGCATACATACTTGTAATTTGACAATATTCATGCACTGAGCAGCGTAATCTG
MYO2AID-F	5'-GAGAATAGTTGACCTTGTTGCCCAACAAGTCGTTCAAGACGGCCAC CGTACGCTGCAGGTCGAC
MYO2AID-R	5'-CTGATGGCGCGAGAAACAAAATTGTACATGAATGCTAAAAAAAGA ATCGATGAATTCGAGCTCG
MYO2AID-check-F	5'-GATTCTAAGGGTCACGAGCA
MYO2AID-check-R	5'-CATAGCAGCAGTATGCGGTT

Supplement Figure1

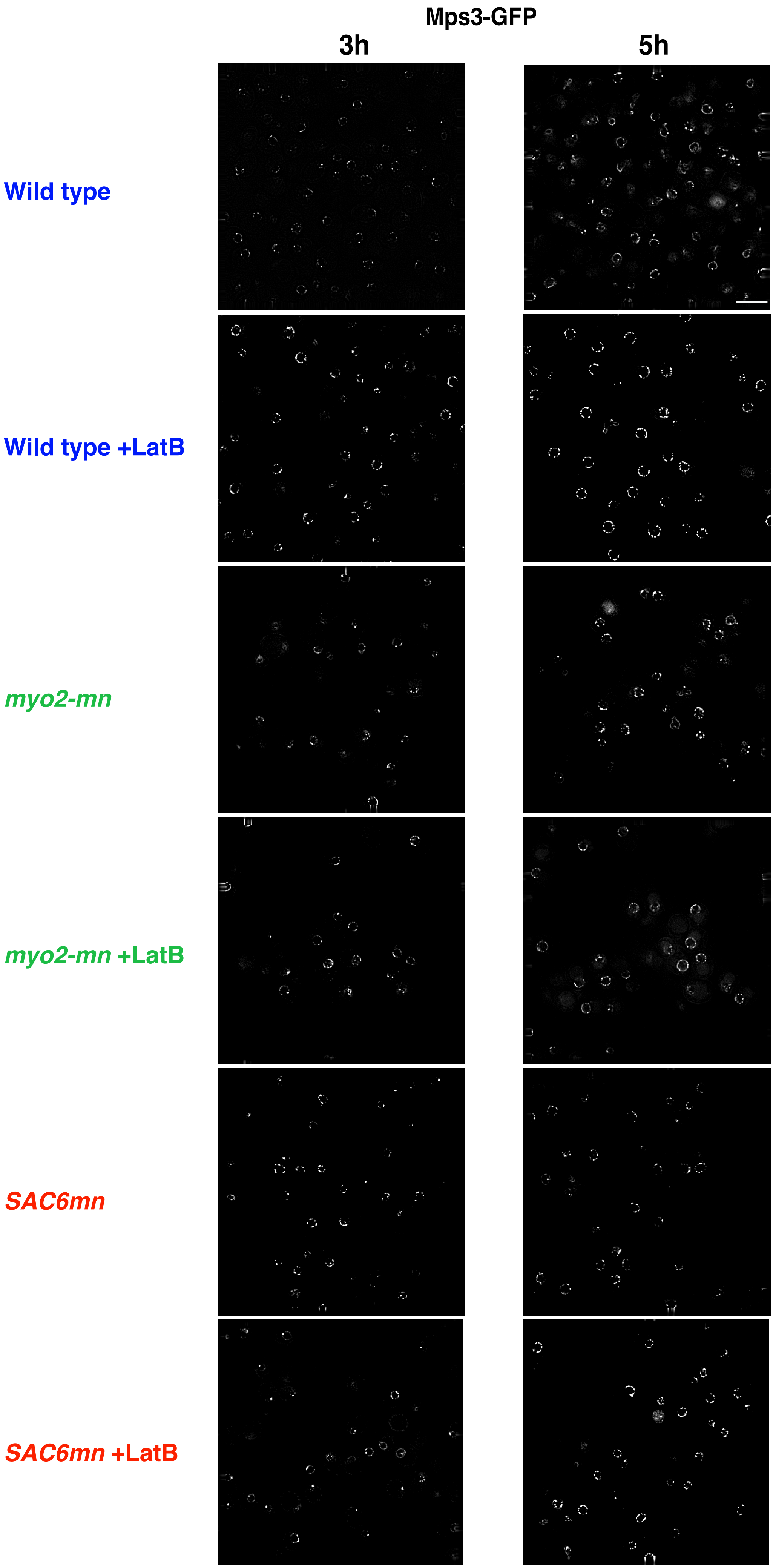

Supplementary Figure 1.

Representative images of cells with Mps3-GFP at 3 and 5 h are shown. The LatB (30 μM) was added at 3 h. Wild type, PRY64-1/64-2; *MYO2mn*, SPY137/139; *SAC6mn*, SPY142/143. Bar indicates 10 μm.

Supplement Figure2

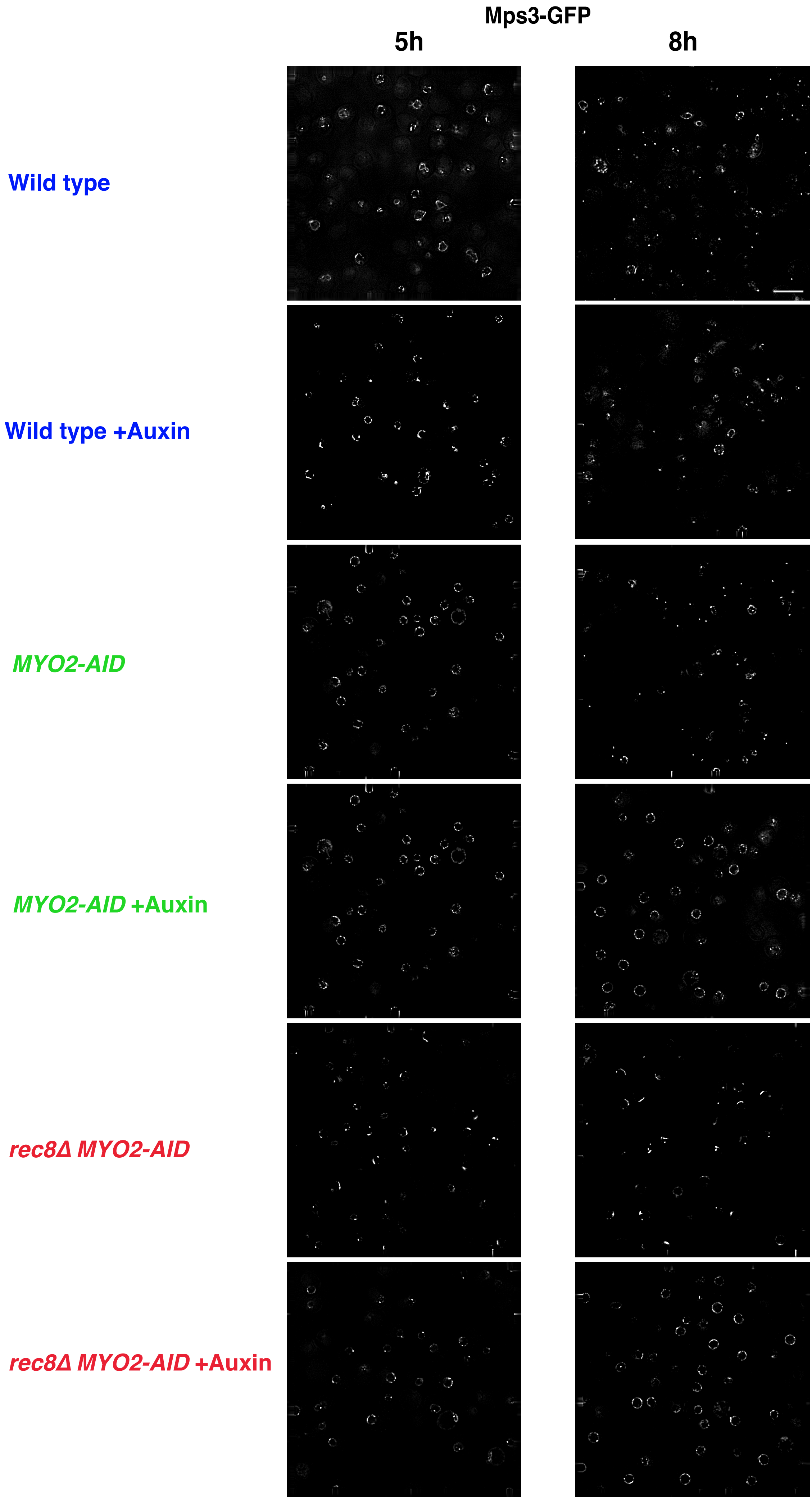

Supplementary Figure 2.

Representative images of cells with Mps3-GFP at 3 and 5 h are shown. The auxin was added at 3 h. Wild type, PRY64-1/64-2; *MYO2-AID*, SPY321/322; *rec8 MYO2-AID*, SPY325/326. The bar indicates 10 μm.

Supplement Figure 3

A

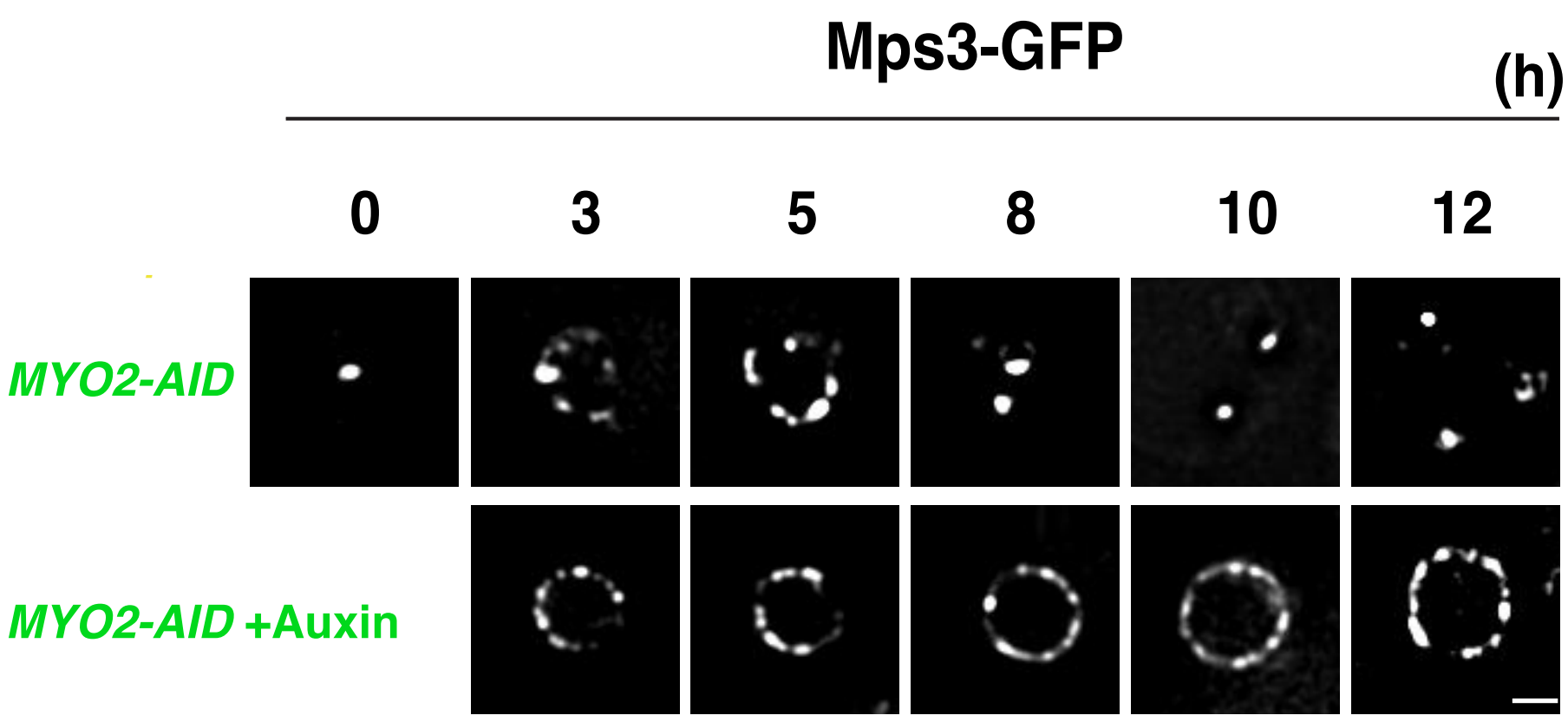

B

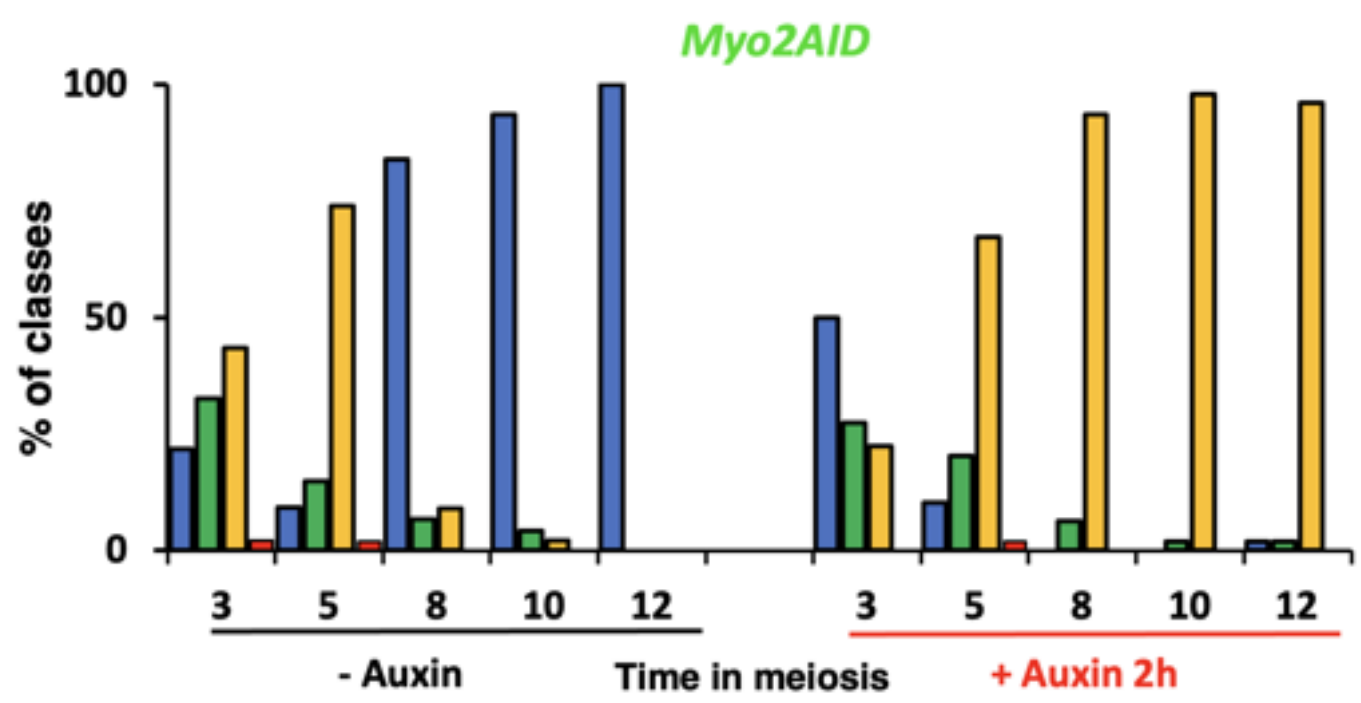

Supplementary Figure 3.

Mps3-GFP localization was analyzed in wild-type (PRY64-1/64-2) and *MYO2-myc-AID* cells (SPY321/322) at various time points during meiosis. Auxin (2 mM) was added at 2 h. Representative images at each time point are shown. The images were taken and processed as shown in Figure 1.
